# Global Stability Analysis of SEIR Model with Holling Type II Incidence Function

**DOI:** 10.1155/2012/826052

**Published:** 2012-10-10

**Authors:** Mohammad A. Safi, Salisu M. Garba

**Affiliations:** ^1^Department of Mathematics, The Hashemite University, Zarqa 13115, Jordan; ^2^Department of Mathematics and Applied Mathematics, University of Pretoria, Pretoria 0002, South Africa

## Abstract

A deterministic model for the transmission dynamics of a communicable disease is developed and rigorously analysed. The model, consisting of five mutually exclusive compartments representing the human dynamics, has a globally asymptotically stable disease-free equilibrium (DFE) whenever a certain epidemiological threshold, known as the basic *reproduction number* (*ℛ*_0_), is less than unity; in such a case the endemic equilibrium does not exist. On the other hand, when the reproduction number is greater than unity, it is shown, using nonlinear Lyapunov function of Goh-Volterra type, in conjunction with the LaSalle's invariance principle, that the unique endemic equilibrium of the model is globally asymptotically stable under certain conditions. Furthermore, the disease is shown to be uniformly persistent whenever *ℛ*_0_ > 1.

## 1. Introduction

 Mathematical models have been widely used to gain insight into the spread and control of emerging and reemerging disease. The dynamics of these models is usually determined by a threshold quantity known as the *basic reproduction number* (denoted by *ℛ*_0_), which is defined as the number of secondary cases generated by an infected individual in a completely susceptible population [1–5]. Characteristically, when *ℛ*_0_ is less than unity, a small influx of infected individuals will not generate large outbreaks, and the disease dies out in time. On the other hand, when *ℛ*_0_ exceeds unity, the disease will persist. A basic epidemic model supports at least two equilibria (a disease-free equilibria and endemic equilibria); *ℛ*_0_ plays important role in the study of equilibria of a model. Several models found in the literature [2, 4–15] have been used to show that when *ℛ*_0_ crosses the threshold, *ℛ*_0_ = 1, a transcritical bifurcation takes place. That is, asymptotic local stability is transferred from the disease-free state to the new (emerging) endemic (positive) equilibria. In some cases, it can be shown that the transfer of asymptotic stability is independent of initial conditions; that is, it is global; see, for instance, [6, 8–12, 16]. Establishing global properties of a dynamical system using Lyapunov function is generally a nontrivial problem. This is owing to the fact that there are no systematic methods for constructing Lyapunov function for infectious disease models with standard incidence rate [17]. The most successful approach to the problem is the direct Lyapunov method which involves the use of quadratic function of the form *ω*(*x*_1_, *x*_2_,…, *x*_*n*_) = ∑_*i*=1_^*n*^(*c*_*i*_/2)(*x*_*i*_ − *x*_i_*) or by using nonlinear Lyapunov function of Goh-Volterra type of the form *ω*(*x*_1_, *x*_2_,…, *x*_*n*_) = ∑_*i*=1_^*n*^(*c*_*i*_/2)(*x*_*i*_ − *x*_i_* − *x*_i_*ln⁡(*x*_*i*_/*x*_i_*)). However, other methods used in establishing global properties of some epidemic models include Dulac's criterion to eliminate the existence of the periodic solution and prove the global stability by the Poincaré Bendixson theorem [18] and those reported in Kamgang and Sallet [19] and Qiao et al. [20]. 

 Let *S*(*t*), *I*(*t*), and *N*(*t*) denote the number of susceptible individuals, infectious individuals, and the total size of the population at time *t*, respectively. Further, let *β*(*N*) be the average number of contacts that is sufficient to transmit infection (effective contact rate). Then, the force of infection, given by *β*(*N*)*I*/*N*, represents the average number of contacts a susceptible individual makes with infectious individuals per unit time. If *β*(*N*) = *βN* (i.e., the contact rate depends on the total population, *N*), then the incidence function *g*_1_(*I*) = *βI* is called *mass action incidence*. If *β*(*N*) = *β* (a constant), then the incidence function *g*_2_(*I*) = *βI*/*N* is called *standard incidence* [4]. These two functions are widely used in modeling the transmission dynamics of the human diseases [1, 21]. Another widely used incidence function is the Holling type II incidence function, given by *g*_3_(*I*) = *βI*/(1 + *ωI*), with *ω* > 0, [22–28].

The nonlinear incidence function of type *g*_3_(*I*) was first introduced by Capasso and Serio [22], in their study of cholera epidemic. The main justification for using such a functional form of the incidence function stems from the fact that the number of effective contacts between infective individuals and susceptible individuals may saturate at high infective levels due to crowding of infective individuals or due to the preventive measures (and behavioral changes) taken by the susceptible individuals in response to the severity of the disease [23, 25–27]. 

 A number of mathematical models have been developed in the literature to gain insights into the transmission dynamics of diseases with subpopulation (compartments) [4, 6–14, 29–31]. The choice of which compartment to include in a model depends on the characteristic of the particular disease being modelled and the purpose of the model [4]. 

 The classical SIR or SIRS model assumed that the disease incubation is negligible so that each susceptible individual (in the *S* class) once infected becomes infectious (and move to *I* class) and later recovers (to move to *R* class) where they acquire permanent or temporary immunity [32]. However, more general models than SIR or SIRS models assumed that susceptible individuals, once infected, first go through the latent period (in the *E* class) before becoming infectious; the resulting models are of SEIR or SEIRS type, depending on whether recovered individuals acquired permanent or temporary immunity [33, 34]. 

 The model considered in this study is based on SEIR (*S*: susceptible, *E*: exposed, *I*: infected, *R*: recovered) where recovered individuals acquire permanent immunity, so that they will not become infected again. This is owing to the fact that for many viral diseases such as measles, smallpox, rubella, HIV/AIDS, and influenza recovered individuals confer lifelong immunity [29, 33]. Huang and Takeuchi [29] study the classical SIR, SIS, SEIR, and SEI models with time delay and a general incidence function. Safi et. al. [13] consider the effect of periodic fluctuations on the transmission dynamics of a communicable disease using SEIRS model, subject to quarantine and isolation; the authors show that adding periodicity to the autonomous model does not alter the threshold dynamics of the model with respect to the control of the disease in the population. Zhang and Ma [35] considered the global dynamics of an SEIR model with saturating contact rate. Korobeinikov [36] established global asymptotic dynamics of SEIR and SIR models with several parallel infectious stages. Li et al. [33] analyzed the global dynamics of an SEIR model with vertical transmission and bilinear incidence. Li and Jin [34] consider global stability of an SEIR epidemic model with infectious force in latent, infected, and immune period. It should be stated, however, that the aforementioned three studies [33, 34, 36] considered mass action (bilinear) incidence to model the infection. This study focuses on the mathematical modeling of the transmission dynamics of an arbitrary disease with educated (counsel) and uneducated infectious stages. 

 This study has three important differences from those reported in [33, 34, 36]. The first is established essential qualitative features of an SEIR model with Holling type II incidence function. The second is that a public health education and counselling are offered to infected individuals (*I*_*u*_). Recent studies further reinforced the widely held belief that one key strategy for preventing and controlling the spread of communicable disease such as HIV (especially in resource-poor nations) is to provide HIV-related public health education and counselling (such as sexual education and awareness of the risk and life-threatening consequences of HIV/AIDS) which would, hopefully, lead to reduction in risky sexual behavior and safer lifestyle within the community [7]. In addition to the above extensions, rigorous qualitative analysis will be provided for the resulting SEIR model. In particular, the paper gives special emphasis on the global asymptotic stability of the disease-free equilibrium and endemic equilibrium. 

 The paper is organized as follows. The model is formulated and analysed for its basic dynamical features in Section 2. The stability analysis of the model is presented in Sections 3 and 4.

## 2. Model Formulation

 The total population at time *t*, denoted by *N*(*t*), is subdivided into five compartments of susceptible (*S*(*t*)), exposed (those who have been infected but are not yet infectious) (*E*(*t*)), uneducated infected individuals (*I*_*u*_(*t*)), educated infected individuals (*I*_*e*_(*t*)), and recovered (*R*(*t*)) individuals, so that

(1) $$\begin{array}{c} N\left(t\right)=S\left(t\right)+E\left(t\right)+I_{u}\left(t\right)+I_{e}\left(t\right)+R\left(t\right). \end{array}$$

The susceptible population is increased by the recruitment of individuals into the population (assumed susceptible), at a rate Π. Susceptible individuals may acquire infection, following effective contact with infected individuals (in the *I*_*u*_ or *I*_*e*_ class) at a rate *λ*(*t*), where

(2) $$\begin{array}{c} \lambda \left(t\right)=\beta \left(\frac{I_{u}}{1+\alpha_{1}I_{u}}+\frac{\eta I_{e}}{1+\alpha_{2}I_{e}}\right). \end{array}$$

In (2), *β* is the effective contact rate (contact capable of leading to infection), while the modification parameter 0 < *η* < 1 accounts for the assumed reduction in disease transmission by educated infected individuals in comparison to uneducated infected individuals in the *I*_*u*_ class. The population of susceptible individuals is further decreased by natural death (at a rate *μ*). Thus, the rate of change of the susceptible population is given by

(3) $$\begin{array}{c} \frac{dS}{dt}=\Pi -\left[\mu +\lambda \left(t\right)\right]S\left(t\right). \end{array}$$

The population of exposed individuals is generated by the infection of susceptible individuals (at the rate *λ*(*t*)). This population is decreased by development of disease symptoms (at a rate *κ*) and natural death (at a rate *μ*), so that

(4) $$\begin{array}{c} \frac{dE}{dt}=\lambda \left(t\right)S\left(t\right)-\left(\kappa +\mu \right)E\left(t\right). \end{array}$$

The population of uneducated infected individuals is generated at the rate *κ*. It is decreased by natural recovery (at a rate *γ*_1_), education (at a rate *σ*), natural death (at the rate *μ*), and disease-induced death (at a rate *δ*_1_). This gives

(5) $$\begin{array}{c} \frac{dI_{u}}{dt}=\kappa E\left(t\right)-\left(\sigma +\gamma_{1}+\mu +\delta_{1}\right)I_{u}\left(t\right). \end{array}$$

The population of educated infected individuals is generated by the education of infected individuals (at the rate *σ*). This population is decreased by recovery (at a rate *γ*_2_), natural death (at the rate *μ*), and disease-induced death (at a rate *δ*_2_ < *δ*_1_). It is assumed that the disease-induced mortality rate of educated infected individuals is low in comparison with uneducated infected individuals. Hence, the rate of change of this population is given by

(6) $$\begin{array}{c} \frac{dI_{e}}{dt}=\sigma I_{u}\left(t\right)-\left(\gamma_{2}+\mu +\delta_{2}\right)I_{e}\left(t\right). \end{array}$$

Finally, the population of recovered individuals is generated by the recovery of uneducated and educated infected individuals (at the rates *γ*_1_ and *γ*_2_, resp.). It is decreased by natural death (at the rate *μ*), so that

(7) $$\begin{array}{c} \frac{dR}{dlt}=\gamma_{1}I_{u}\left(t\right)+\gamma_{2}I_{e}\left(t\right)-\mu R\left(t\right). \end{array}$$

Thus, the model for the transmission dynamics of an infectious disease in the presence of educated (counsel) infected individuals is given by the following nonlinear system of differential equations:

(8) dSdt=Π−[μ+λ(t)]S(t),dEdt=λ(t)S(t)−[μ+κ]E(t),dIudt=κE(t)−[μ+σ+γ1+δ1]Iu(t),dIedt=σIu(t)−[μ+γ2+δ2]Ie(t),dRdlt=γ1Iu(t)+γ2Ie(t)−μR(t).

The model (8) extends some SEIR models in the literature such as those in [33, 34, 36] by

- replacing the mass action incidence function with Holling type II incidence function,
- splitting the compartment of infected individuals into educated (counsel) and uneducated (noncounsel) infected individuals thereby allowing time-varying infection rate. The epidemiological implication of this assumption is that educated infected individuals transmit the disease at a reduced rate (0 < *η* < 1) in comparison to the uneducated infected individuals (due to behavioral changes). For instance, In Zambia, the decline in HIV incidence since early 1990s is attributed to behavioral changes [37]. Public health education campaigns have also been successfully implemented in numerous countries and communities, such as Uganda, Thailand, Zambia, and the US gay community [38, 39]. Between 1991 and 1998, HIV prevalence dramatically declined in Uganda from 21 to 9.8% (with a corresponding reduction in nonregular sexual partners by 65% coupled with greater levels of awareness about HIV/AIDS [38]. The Ugandan programme fostered community mobilization towards change in risky behavior, without increasing stigma [40].

Further, unlike in the aforementioned modelling studies, detailed rigorous mathematical analysis of the model (8) will be provided. 

### 2.1. Basic Properties

 Since the model (8) monitors human populations, all its associated parameters are nonnegative. Further, the following nonnegativity result holds.

**Theorem 1**

The variables of the model (8) are nonnegative for all the time. In other words, solutions of the model system (8) with positive initial data will remain positive for all time *t* > 0.

**Proof**

Let *t*_1_ = sup⁡{*t* > 0 : *S* > 0, *E* > 0, *I*_*u*_ > 0, *I*_*e*_ > 0, *R* > 0 ∈ [0, *t*]}. Thus, *t*_1_ > 0. It follows from the first equation of the system (8) that

(9) $$\begin{array}{c} \frac{dS}{dt}=\Pi -\lambda \left(t\right)S\left(t\right)-\mu S\left(t\right)\geq \Pi -\left(\lambda +\mu \right)S\left(t\right), \end{array}$$

which can be rewritten as

(10) $$\begin{array}{c} \frac{d}{dt}\left[S\left(t\right)\exp\left\{\mu t+\int_{0}^{t}\lambda \left(\tau \right)d\tau \right\}\right]\geq \Pi \exp\left\{\mu t+\int_{0}^{t}\lambda \left(\tau \right)d\tau \right\}. \end{array}$$

Hence,

(11) S(t1)exp⁡{μt1+∫0tλ(τ)dτ}−S(0) ≥∫0t1Πexp⁡{μy+∫0yλ(τ)dτ}dy,

so that

(12) S(t)≥S(0)exp⁡{−μt1−∫0t1λ(τ)dτ}   ×[exp⁡{−μt1−∫0t1λ(τ)dτ}]   ×∫0t1Πexp⁡{μy+∫0yλ(τ)dτ}dy>0.

Similarly, it can be shown that *E* > 0, *I*_*u*_ > 0, *I*_*e*_ > 0 and *R* > 0, for all time *t* > 0.

The previous result can also be established using the method in of [41, Appendix A].

 We claim the following result.

**Lemma 2**

The closed set

(13) $$\begin{array}{c} 𝒟=\left\{\left(S,E,I_{u},I_{e},R\right)\in {\mathbb{R}}_{+}^{5}:S+E+I_{u}+I_{e}+R\leq \frac{\Pi }{\mu }\right\} \end{array}$$

is positively invariant.

**Proof**

Adding all the equations of the model (8) gives,

(14) $$\begin{array}{c} \frac{dN}{dt}=\Pi -\mu N-\left(\delta_{1}I_{u}+\delta_{2}I_{e}\right). \end{array}$$

Since *dN*/*dt* ≤ Π − *μN*, it follows that *dN*/*dt* ≤ 0 if *N* ≥ Π/*μ*. Thus, a standard comparison theorem [42] can be used to show that *N* ≤ *N*(0)*e* ^−*μt*^ + (Π/*μ*)(1 − *e* ^−*μt*^). In particular, *N*(*t*) ≤ Π/*μ* if *N*(0) ≤ Π/*μ*. Thus, the region *𝒟* is positively invariant. Further, if *N*(0) > Π/*μ*, then either the solution enters *𝒟* infinite time or *N*(*t*) approaches Π/*μ* asymptotically. Hence, the region *𝒟* attracts all solutions in ℝ_+_^5^.

Since the region *𝒟* is positively invariant, it is sufficient to consider the dynamics of the flow generated by the model (8) in *𝒟*, where the usual existence, uniqueness, continuation results hold for the system [10].

## 3. Local Stability of Disease-Free Equilibrium (DFE)

 The DFE of the model (8) is given by

(15) $$\begin{array}{c} {ℰ}_{0}=\left(S^{*},E^{*},I_{u}^{*},I_{e}^{*},R^{*}\right)=\left(\frac{\Pi }{\mu },0,0,0,0\right). \end{array}$$

The local stability of *ℰ*_0_ will be explored using the next generation operator method [43, 44]. Using the notation in [44], the nonnegative matrix, *F*, of the new infection terms and the *M*-matrix, *V*, of the transition terms associated with the model (8) are given, respectively, by

(16) $$\begin{array}{c} F=\left(\begin{array}{ccc} 0 & \frac{\beta \Pi }{\mu } & \frac{\eta \beta \Pi }{\mu }\\ 0 & 0 & 0\\ 0 & 0 & 0 \end{array}\right),\\ V=\left(\begin{array}{cccc} \mu +\kappa & 0 & 0 & \\ -\kappa & \mu +\sigma +\gamma_{1}+\delta_{1} & 0 & \\ 0 & -\sigma & \mu +\gamma_{2}+\delta_{2} & \end{array}\right). \end{array}$$

It follows that the *control reproduction number* [4, 21], denoted by *ℛ*_0_ = *ρ*(*FV* ^−1^), where *ρ* is the spectral radius, is given by

(17) $$\begin{array}{c} {ℛ}_{0}=\frac{\beta \Pi \kappa \left(k_{3}+\eta \sigma \right)}{\mu k_{1}k_{2}k_{3}}, \end{array}$$

where

(18) $$\begin{array}{c} k_{1}=\mu +\kappa ,\;k_{2}=\mu +\sigma +\gamma_{1}+\delta_{1},\;\;k_{3}=\mu +\gamma_{2}+\delta_{2}. \end{array}$$

Using in [44, Theorem 2], the following result is established.

**Lemma 3**

The DFE of the model (8), given by (15), is locally asymptotically stable (LAS) if *ℛ*_0_ < 1 and unstable if *ℛ*_0_ > 1.

The quantity *ℛ*_0_ measures the average number of new infections generated by a single infected individual in a population.  Lemma 3 implies that the disease can be eliminated from the community (when *ℛ*_0_ < 1) if the initial sizes of the subpopulations of the model are in the basin of attraction of the DFE (*ℰ*_0_). To ensure that disease elimination is independent of the initial sizes of sub-populations, it is necessary to show that the DFE is globally asymptotically stable (GAS) if *ℛ*_0_ < 1. This is explored below.

### 3.1. Global Stability of DFE

**Theorem 4**

The DFE of the model (8), given by (15), is GAS in *𝒟* whenever *ℛ*_0_ ≤ 1.

**Proof**

Consider the following Lyapunov function:

(19) $$\begin{array}{c} ℱ=\left(\frac{\kappa \left(k_{3}+\eta \sigma \right)}{\eta k_{1}k_{2}}\right)E+\left(\frac{k_{3}+\eta \sigma }{\eta k_{2}}\right)I_{u}+I_{e}, \end{array}$$

with Lyapunov derivative (where a dot represents differentiation with respect to time) given by

(20) ℱ˙=(κ(k3+ησ)ηk1k2)E˙+(k3+ησηk2)I˙u+I˙e=(κ(k3+ησ)ηk1k2)[βS(Iu1+α1Iu+ηIe1+α2Ie)−k1E]+(k3+ησηk2)[κE−k2Iu]+σIu−k3Ie≤(κ(k3+ησ)ηk1k2)[βΠμ(Iu+ηIe)−k1E]+(k3+ησηk2)[κE−k2Iu]+σIu−k3Ie=βΠκ(k3+ησ)μηk1k2(Iu+ηIe)+(σ−k3+ηση)Iu−k3Ie=k3η(ℛ0−1)(Iu+ηIe).

Since all the parameters and variables of the model (8) are nonnegative (Theorem 1), it follows that $\dot{ℱ}\leq 0$ for *ℛ*_0_ ≤ 1 with $\dot{ℱ}=0$ if and only if *E* = *I*_*u*_ = *I*_*e*_ = 0. Hence, *ℱ* is a Lyapunov function on *𝒟*. Therefore, the largest compact invariant subset of the set where $\dot{ℱ}=0$ is the singleton {(*E*, *I*_*u*_, *I*_*e*_) = (0,0, 0)}. Thus, it follows, by the LaSalle's invariance principle [18, 45], that

(21) $$\begin{array}{c} \left(E,I_{u},I_{e}\right)\to \left(0,0,0\right)\;\;\;\;\text{as}\;\;t\to \infty . \end{array}$$

Since limsup⁡_*t*→*∞*_ *I*_*u*_ = 0 and limsup⁡_*t*→*∞*_ *I*_*e*_ = 0 (from (21)), it follows that, for sufficiently small ϖ* > 0, there exist constants *M*_1_ > 0 and *M*_2_ > 0 such that limsup⁡_*t*→*∞*_ *I*_*u*_ ≤ ϖ* for all *t* > *M*_1_ and limsup⁡_*t*→*∞*_ *I*_*e*_ ≤ ϖ* for all *t* > *M*_2_. Hence, it follows from the last equation of the model (8) that, for *t* > max⁡{*M*_1_, *M*_2_},

(22) $$\begin{array}{c} \dot{R}\leq \gamma_{1}\varpi^{*}+\gamma_{2}\varpi^{*}-\mu R. \end{array}$$

Thus, by comparison theorem [42],

(23) $$\begin{array}{c} R^{\infty }=\underset{t\to \infty }{\mathrm{limsup}}R\leq \frac{\gamma_{1}\varpi^{*}+\gamma_{2}\varpi^{*}}{\mu }, \end{array}$$

so that, by letting ϖ* → 0,

(24) $$\begin{array}{c} R^{\infty }=\underset{t\to \infty }{\mathrm{limsup}}R\leq 0. \end{array}$$

Similarly (by using liminf⁡_*t*→*∞*_ *I*_*u*_ = 0 and liminf⁡_*t*→*∞*_ *I*_*e*_ = 0), it can be shown that

(25) $$\begin{array}{c} R_{\infty }=\underset{t\to \infty }{\mathrm{liminf}}R\geq 0. \end{array}$$

Thus, it follows from (24) and (25) that

(26) $$\begin{array}{c} R_{\infty }\geq 0\geq R^{\infty }. \end{array}$$

Hence,

(27) $$\begin{array}{c} \underset{t\to \infty }{\lim}R=0. \end{array}$$

Similarly, it can be shown that

(28) $$\begin{array}{c} \underset{t\to \infty }{\lim}S\left(t\right)=\frac{\Pi }{\mu }. \end{array}$$

Thus, by combining (21), (27), and (28), it follows that every solution of the equations of the model (8), with initial conditions in *𝒟*, approaches *ℰ*_0_ as *t* → *∞* (for *ℛ*_0_ < 1).

The previous result shows that the disease can be eliminated from the community if the associated reproduction number of the model is less than unity.

## 4. Existence and Stability for Endemic Equilibrium Point

 In this section, the possible existence and stability of endemic (positive) equilibria of the model (8) (i.e., equilibria where at least one of the infected components of the model is nonzero) will be explored. 

### 4.1. Existence of Endemic Equilibrium Point (EEP)

 First of all, the persistence of the disease in the population will be investigated below. The model system (8) is said to be uniformly persistent if there exists a constant *c* such that any solution (*S*(*t*), *E*(*t*), *I*_*u*_(*t*), *I*_*e*_(*t*), *R*(*t*)) satisfies ([31, 46])

(29) $$\begin{array}{c} \underset{t\to \infty }{\mathrm{liminf}}S\left(t\right)\geq c,\;\underset{t\to \infty }{\mathrm{liminf}}E\left(t\right)\geq c,\;\underset{t\to \infty }{\mathrm{liminf}}I_{u}\left(t\right)\geq c,\\ \underset{t\to \infty }{\mathrm{liminf}}I_{e}\left(t\right)\geq c,\;\underset{t\to \infty }{\mathrm{liminf}}R\left(t\right)\geq c, \end{array}$$

provided that (*S*(0), *E*(0), *I*_*u*_(0), *I*_*e*_(0), *R*(0)) ∈ *𝒟*° (the interior of the region *𝒟*).

**Theorem 5**

System (8) is uniformly persistent in *𝒟*° if and only if *ℛ*_0_ > 1.

**Proof**

The theorem can be proved by using the approach used to prove Proposition 3.3 of [32], by applying a uniform persistence result in [46] and noting that the DFE of the model (8) is unstable whenever *ℛ*_0_ > 1 (Lemma 3).

When *ℛ*_0_ > 1, it follows (from Theorem (3)) that model (8) is uniformly persistent; by using in [47, Theorem 2.8.6] and in [42, Theorem D.3] it follows that the model (8) has at least one endemic equilibrium in *𝒟*°. Thus the following result is established.

**Lemma 6**

The model (2) has at least one endemic equilibrium, given by *ℰ*_1_ = (S**, E**, *I*_u_**, *I*_e_**, R**), whenever *ℛ*_0_ > 1.

The uniqueness of the endemic equilibrium will be investigated at the end of the next subsection. 

### 4.2. Global Stability of Endemic Equilibrium for Special Case

 Here, the global asymptotic stability property of the endemic equilibrium of the model (8) is given for a special case when educated (counsel) infected individuals do not transmit infection (*η* = 0). The model (8), with *η* = 0, then reduces to:

(30) dSdt=Π−[μ+βf(Iu)]S(t),dEdt=βf(Iu)S(t)−[μ+κ]E(t),dIudt=κE(t)−[μ+σ+γ1+δ1]Iu(t),dIedt=σIu(t)  −[μ+γ2+δ2]Ie(t)dRdt=γ1Iu(t)+γ2Ie(t)−μR(t),

where

(31) $$\begin{array}{c} f\left(I_{u}\right)=\frac{I_{u}}{1+\alpha_{1}I_{u}}. \end{array}$$

The reproduction number of the model (30) is given by

(32) $$\begin{array}{c} {ℛ}_{0r}={ℛ}_{0}{|}_{\eta =0}=\frac{\beta \Pi \kappa }{\mu k_{1}k_{2}}. \end{array}$$

We claim the following result.

**Theorem 7**

The endemic equilibrium of the reduced model, given by (30), is GAS in *𝒟*° if *ℛ*_0*r*_ > 1. Thus it is unique.

**Proof**

Consider the reduced model, given by (30). Let *ℛ*_0*r*_ > 1, so that the associated endemic equilibrium exists. Further, consider the following nonlinear Lyapunov function:

(33) ℱ=S−S∗∗−S∗∗ln⁡(SS∗∗)+E−E∗∗−E∗∗ln⁡(EE∗∗)+k1κ[Iu−Iu∗∗−Iu∗∗ln⁡(IuIu∗∗)]

with Lyapunov derivative

(34) ℱ˙=S˙−S∗∗SS˙+E˙−E∗∗EE˙+k1κ(I˙u−Iu∗∗IuI˙u), =Π−βSf(Iu)−μS−S∗∗S(Π−βSf(Iu)−μS)    +βSf(Iu)−k1E−E∗∗E(βSf(Iu)−k1E)  +k1κ[κE−k2Iu−Iu∗∗Iu(κE−k2Iu)] =Π(1−S∗∗S)−μS(1−S∗∗S)  +βS∗∗f(Iu)−k1k2κIu−E∗∗βSf(Iu)E  +k1E∗∗−k1Iu∗∗EIu+k1k2Iu∗∗κ.

It can be shown from (30) that, at endemic steady state,

(35) $$\begin{array}{c} \Pi =\left[\mu +\beta f\left(I_{u}^{**}\right)\right]S^{**},\\ k_{1}=\frac{\beta f\left(I_{u}^{**}\right)S^{**}}{E^{**}},\\ k_{2}=\frac{\kappa E^{**}}{I_{u}^{**}}. \end{array}$$

Using the relations ([Disp-formula EEq13]) in (34) gives

(36) ℱ˙=μS∗∗(2−S∗∗S−SS∗∗)−(S∗∗)2f(Iu∗∗)S+βS∗∗f(Iu∗∗)+βS∗∗f(Iu)−βS∗∗f(Iu∗∗)IuIu∗∗−E∗∗βSf(Iu)E+βS∗∗f(Iu∗∗)−βS∗∗Iu∗∗f(Iu∗∗)EIuE∗∗+βS∗∗f(Iu∗∗).

Adding and subtracting   βS**f(*I*_u_**) and βS**f ^2^(*I*_u_**)*I*_*u*_/*f*(*I*_*u*_)*I*_u_**   in (36) gives

(37) ℱ˙=μS∗∗(2−S∗∗S−SS∗∗)+βS∗∗f(Iu∗∗)×(4−S∗∗S−E∗∗Sf(Iu)ES∗∗f(Iu∗∗)−Iu∗∗EIuE∗∗−f(Iu∗∗)Iuf(Iu)Iu∗∗)+βS∗∗f(Iu)−βS∗∗f(Iu∗∗)IuIu∗∗−βS∗∗f(Iu∗∗)+βS∗∗f2(Iu∗∗)Iuf(Iu)Iu∗∗.

Rewriting the last four terms of (37) in terms of Π − μS** (by using the relations in ([Disp-formula EEq13])) gives

(38) ℱ˙=μS∗∗(2−S∗∗S−SS∗∗)+βS∗∗f(Iu∗∗)×(4−S∗∗S−E∗∗Sf(Iu)ES∗∗f(Iu∗∗)−Iu∗∗EIuE∗∗−f(Iu∗∗)Iuf(Iu)Iu∗∗)+(Π−μS∗∗)f(Iu)f(Iu∗∗)−(Π−μS∗∗)IuIu∗∗−(Π−μS∗∗)+(Π−μS∗∗)f(Iu∗∗)Iuf(Iu)Iu∗∗=μS∗∗(2−S∗∗S−SS∗∗)+βS∗∗f(Iu∗∗)×(4−S∗∗S−E∗∗Sf(Iu)ES∗∗f(Iu∗∗)−Iu∗∗EIuE∗∗−f(Iu∗∗)Iuf(Iu)Iu∗∗)+(Π−μS∗∗)[f(Iu)f(Iu∗∗)−IuIu∗∗−1+f(Iu∗∗)Iuf(Iu)Iu∗∗]=μS∗∗(2−S∗∗S−SS∗∗)+βS∗∗f(Iu∗∗)×(4−S∗∗S−E∗∗Sf(Iu)ES∗∗f(Iu∗∗)−Iu∗∗EIuE∗∗−f(Iu∗∗)Iuf(Iu)Iu∗∗)+(Π−μS∗∗)Iuf(Iu)[f(Iu)Iu−f(Iu∗∗)Iu∗∗]×[f(Iu)f(Iu∗∗)−1].

Since *f*(*I*_*u*_) is an increasing function and S** ≤ Π/*μ*, it follows that

(39) $$\begin{array}{c} \left(\Pi -\mu S^{**}\right)\frac{I_{u}}{f\left(I_{u}\right)}\left[\frac{f\left(I_{u}\right)}{I_{u}}-\frac{f\left(I_{u}^{**}\right)}{I_{u}^{**}}\right]\left[\frac{f\left(I_{u}\right)}{f\left(I_{u}^{**}\right)}-1\right]\leq 0, \end{array}$$

finally, since the arithmetic mean exceeds the geometric mean, then

(40) $$\begin{array}{c} \left(2-\frac{S^{**}}{S}-\frac{S}{S^{**}}\right)\leq 0, \end{array}$$

(41) $$\begin{array}{c} \left(4-\frac{S^{**}}{S}-\frac{E^{**}Sf\left(I_{u}\right)}{ES^{**}f\left(I_{u}^{**}\right)}-\frac{I_{u}^{**}E}{I_{u}E^{**}}-\frac{f\left(I_{u}^{**}\right)I_{u}}{f\left(I_{u}\right)I_{u}^{**}}\right)\leq 0. \end{array}$$

Further, since all the model parameters are nonnegative, it follows that $\dot{ℱ}\leq 0$ for *ℛ*_0*r*_ > 1 with $\dot{ℱ}=0$ if and only if *S* = S**, *E* = E**, *I*_*u*_ = *I*_u_**. Hence, *ℱ* is a Lyapunov function on *𝒟*°. Therefore, the largest compact invariant subset of the set where $\dot{ℱ}=0$ is the singleton {(*S*, *E*, *I*_*u*_) = (S**, E**, *I*_u_**)}. Thus, it follows, by the laSalle's invariance Principle [18, 45], that *S*(*t*) → S**,*E*(*t*) → E** and *I*_*u*_(*t*) → *I*_u_** as *t* → *∞*. Since limsup⁡_*t*→*∞*_ *I*_*u*_ = *I*_u_**, it follows that, for sufficiently small *ϵ* > 0, there exist constants *T*_1_ > 0 such that limsup⁡_*t*→*∞*_ *I*_*u*_ ≤ *I*_u_** + *ϵ* for all *t* > *T*_1_. It follows from the fourth equation of (30) that, for *t* > *T*_1_,

(42) $$\begin{array}{c} {\dot{I}}_{e}\left(t\right)\leq \sigma \left(I_{u}^{**}+\epsilon \right)-k_{3}I_{e}. \end{array}$$

Thus, by comparison theorem [42],

(43) $$\begin{array}{c} I_{e}^{\infty }=\underset{t\to \infty }{\mathrm{limsup}}\leq \frac{\sigma \left(I_{u}^{**}+\epsilon \right)}{k_{3}}. \end{array}$$

Hence, by letting *ϵ* → 0, we have

(44) $$\begin{array}{c} I_{e}^{\infty }=\underset{t\to \infty }{\mathrm{limsup}}\leq \frac{\sigma I_{u}^{**}}{k_{3}}. \end{array}$$

Similarly, by using liminf⁡_*t*→*∞*_ *I*_*u*_(*t*) = *I*_u_**, it can be shown that

(45) $$\begin{array}{c} {I_{e}}_{\infty }=\underset{t\to \infty }{\mathrm{liminf}}\geq \frac{\sigma I_{u}^{**}}{k_{3}}. \end{array}$$

Thus, it follows from (44) and (45) that

(46) $$\begin{array}{c} {I_{e}}_{\infty }\geq \frac{\sigma I_{u}^{**}}{k_{3}}\geq I_{e}^{\infty }. \end{array}$$

Hence, lim⁡_*t*→*∞*_ *I*_*e*_(*t*) = *σI*_u_**/*k*_3_ = *I*_e_**. In a similar way, it can be shown that lim⁡_*t*→*∞*_ *R*(*t*) = R**. Thus, every solution to the equations of the reduced model, with initial condition in *𝒟*°, approaches the endemic equilibrium of the reduced system (30) as *t* → *∞* for *ℛ*_0*r*_ > 1. This shows that the endemic equilibrium is unique. 

## 5. Conclusions

 In this paper, an SEIR epidemic model with a nonlinear (Holling type II) incidence rate is designed and analysed. Some of the theoretical and epidemiological findings of the study are as follows.

- The model (8) has a locally stable disease-free equilibrium whenever the *associated reproduction number* is less than unity.
- The DFE of the model (8) is shown to be globally asymptotically stable when *ℛ*_0_ < 1.
- The model (8) is uniformly persistent in *𝒟*° if and only if *ℛ*_0_ > 1.
- The endemic equilibrium of the reduced model (8), with *η* = 0, is shown to be globally asymptotically stable, when it exists.
